# Statistical Optimization of the Physico-Chemical Parameters for Pigment Production in Submerged Fermentation of *Talaromyces albobiverticillius* 30548

**DOI:** 10.3390/microorganisms8050711

**Published:** 2020-05-11

**Authors:** Mekala Venkatachalam, Alain Shum-Chéong-Sing, Laurent Dufossé, Mireille Fouillaud

**Affiliations:** 1 Laboratoire de Chimie et Biotechnologie des Produits Naturels-EA 2212, Université de la Réunion, 15 Avenue René Cassin, CS 92003, CEDEX 9, F-97744 Saint-Denis, Ile de la Réunion, France; mekalavenkat@gmail.com (M.V.); alain.shum@univ-reunion.fr (A.S.-C.-S.); laurent.dufosse@univ-reunion.fr (L.D.); 2 Ecole Supérieure d’Ingénieurs Réunion Océan Indien-ESIROI, 2 Rue Joseph Wetzell, F-97490 Sainte-Clotilde, Ile de la Réunion, France

**Keywords:** *Talaromyces albobiverticillius* 30548, submerged fermentation, pigments, biomass, optimization, Box–Behnken experimental design, response surface modeling

## Abstract

*Talaromyces albobiverticillius* 30548 is a marine-derived pigment producing filamentous fungus, isolated from the La Réunion island, in the Indian Ocean. The objective of this study was to examine and optimize the submerged fermentation (SmF) process parameters such as initial pH (4–9), temperature (21–27 °C), agitation speed (100–200 rpm), and fermentation time (0–336 h), for maximum production of pigments (orange and red) and biomass, using the Box–Behnken Experimental Design and Response Surface Modeling (BBED and RSM). This methodology allowed consideration of multifactorial interactions between a set of parameters. Experiments were carried out based on the BBED using 250 mL shake flasks, with a 100 mL working volume of potato dextrose broth (PDB). From the experimental data, mathematical models were developed to predict the pigments and biomass yields. The individual and interactive effects of the process variables on the responses were also investigated (RSM). The optimal conditions for maximum production of pigments and biomass were derived by the numerical optimization method, as follows—initial pH of 6.4, temperature of 24 °C, agitation speed of 164 rpm, and fermentation time of 149 h, respectively.

## 1. Introduction

In recent years, development of alternative sources for the production of natural pigments has been focused on overcoming the unlimited usage of synthetic pigments, which are found to be hazardous to the human health and environment [1]. Natural colorants obtained from plants, animals, and microbial sources are alternatives to synthetic pigments [2,3]. Nowadays, the shift towards natural colorants is of great interest worldwide and is gaining its industrial importance in food, cosmetics, pharmaceuticals, paint, or dying applications. Microorganisms including bacteria, algae, yeasts, and filamentous fungi from marine or terrestrial origins are capable of producing natural dyes, which pave the way to develop the industrial production of natural compounds [4,5,6]. Indeed, microbial fermentations present the crucial advantages of independence from geographic or climatic constraints. Additionally, massive production of pigments using bioreactors can be totally controlled and optimized. The use of fungi for the production of commercially important products has been increasing rapidly over the past decades [4,7]. Taxonomically, it represents a large group, often prevailing in seawater, rocks, sediments, sand, soils, and diverse marine macro-organisms [5]. In general, the colorants produced by fungi are associated with increased yields, and huge array of compounds due to the abundance and vast diversity of fungal strains in the environment [8]. Majority of the pigments produced by fungi are quinones, flavonoids, melanins, and azaphilones, which belong to the aromatic polyketide chemical group [9,10,11] and have been widely described for their medicinal uses or potential utilization as dyes [12,13]. More specifically, filamentous fungi are considered to be a promising source of natural pigments. Moreover, some of the produced molecules also possess bioactive properties like anti-cancer, immunomodulatory, anti-proliferative, antibiotic properties, and so on [14].

Fungi isolated from the marine environments reveal a plethora of known or new compounds with still relatively unexplored bioactivities [15,16,17]. Marine fungi of several genera, such as *Aspergillus, Cladosporium, Eurotium, Monascus, Penicillium*, *Talaromyces*, and *Trichoderma* are potent pigment producers of different classes, with wide range of hues [18,19,20,21,22]. For fungal pigment production, submerged fermentation (SmF) appears to be a convenient and economical fermentation method. Indeed, SmF provides the space to produce pigments, owing to their deep commitment to sophisticated control devices and easy monitoring methods during production [23,24,25]. Besides this, SmF processes can also be affected by various abiotic factors, such as pH of the medium, temperature, agitation speed (gas diffusion), and fermentation time. The mentioned abiotic factors create a strong pressure on the pattern of fungal growth that brings out the interesting secondary metabolites like pigments [26,27,28,29].

Over the past few years, a number of studies identified that several species of *Penicillium* and *Talaromyces* produce *Monascus*-like red pigments without the co-production of mycotoxin citrinin, which is produced in *Monascus spp.* fermentations [30]. Some species of *Talaromyces* secrete large amounts of red pigment, notably species such as *Talaromyces purpurogenus*, *T. albobiverticillius*, *T. marneffei*, and *T*. *minioluteus* (often referenced in the literature under earlier *Penicillium* names). Isolates identified as *T. purpurogenus* have been considered to be industrially interesting for their red pigment production. Unfortunately, the pigment production comes along with the co-production of mycotoxins such as rubratoxin a and b and luteoskyrin. Whereas, a novel strain, *Talaromyces atroroseus* CBS 133442 produces diffusible red pigments, without any mycotoxin production [19]. Certain strains of *Talaromyces albobiverticillius* were studied and proposed to produce large amount of red pigments, thus, can be used for coloring food [31]. However, considering *T. albobiverticillius* 30548, collecting large amount of pigments is a real challenge. First, at a lab scale it allows the purification, characterization, and structural elucidation of all molecules produced, including undesirable compounds, if any. In a second step, optimizing the production of compounds of interest is crucial to determining the ultimate potential of the fungus, for a successful production of pigments at an industrial scale.

The present study investigated and optimized the SmF process crucial parameters such as initial pH (4–9), temperature (21–27 °C), agitation speed (100–200 rpm), and fermentation time (24–336 h) for the maximum production of pigments (orange and red) and biomass by *Talaromyces albobiverticillius* 30548. To achieve this goal, Box–Behnken Experimental Design (BBED) and response surface modeling (RSM) were used. BBED is a methodology based on a three interlocking 2^2^ factorial experimental design. It is also described as a spherical revolving surface surrounding a cube constructed from the responses of the center and middle points of the experimental design [32,33,34,35,36,37]. This experimental design allows consideration of the multifactorial interactions between a set of crucial parameters. Derived from this mathematical approach, it is possible to optimize the culture conditions for maximum production of pigments or biomass and its interactive effects [38]. This is the first report to investigate and optimize the SmF process parameters by BBED and RSM, for maximal pigment and biomass production, using the marine-derived *T. albobiverticillius* 30548.

## 2. Materials and Methods 

### 2.1. Microorganism and Maintenance

The filamentous fungus used in this study was isolated from the sediments collected from the marine environment of the La Réunion island, Indian Ocean (GPS coordinates: 21°06′22.11″ S, 55° 14′15.78″ E) [39]. From taxonomic identification of the fungal biodiversity center (Westerdijk Fungal Biodiversity Institute, Utrecht, The Netherlands), the fungus was identified as *Talaromyces albobiverticillius* and named as *T. albobiverticillius* 30548 (GenBank accession number MK937814). The isolated fungus was cultured in potato dextrose agar plates (PDA, BD-Difco, Sparks, NV, USA) and maintained at 4 °C, as well as sub-cultured at regular intervals for the experiments.

### 2.2. Preparation of Pre-Culture

Laboratory grade potato dextrose broth (PDB) (BD-Difco)) was used to prepare the seed medium. For inoculum preparation, 100 mg of mycelial spores was scrapped from actively grown culture on potato dextrose agar petri plate (PDA), (7 days old) (composition: PDB added with 2% Bacto agar (BD-Difco) and transferred into 250 mL Erlenmeyer flask containing 80 mL of sterilized PDB medium (pre-culture). The flasks were incubated at 25 °C for 48 h in an orbital shaker, at 200 rpm (Multitron Pro, Infors HT, Bottmingen, Switzerland) before being transferred to the main fermentation medium [19].

### 2.3. Fermentation Conditions

After 48 h of growth, the pre-culture broth was centrifuged at 8000 rpm for 6 min at room temperature (Sigma 4K15, Sigma Laborzentrifugen GmbH, Osterode am Harz, Germany) to separate the mycelia and the culture filtrate. The harvested mycelium (200 mg) was transferred as seed inoculum into a 500 mL Erlenmeyer flask containing 200 mL of sterile fermentation medium (PDB). Twenty-nine (29) series of submerged fermentations were carried out according to the experimental design (BBED) presented in Table 1. The BBD matrix (Table 2) allowed to fix a set of selected values for initial pH, temperature, agitation speed, and fermentation time, for each fermentation. The flasks were incubated in a controlled orbital shaker with the fixed conditions (Multitron Pro, Infors HT, Bottmingen, Switzerland) during the whole culture period. All experiments were performed in triplicates for each condition. At predefined intervals of 24, 168, 336 h (Table 1), fermented broth samples were taken out from each culture flask, in order to measure the concentration of orange and red pigments, as well as the increase in biomass weight.

### 2.4. Spectrophotometric Quantification of Extracellular Pigments 

**Figure A1:**
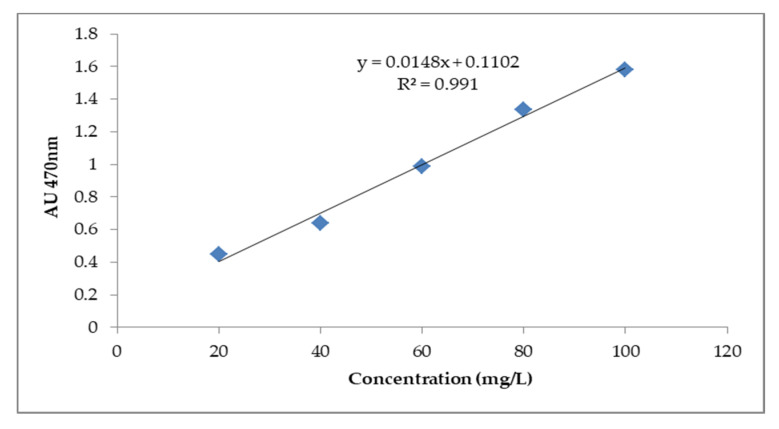
Standard curve for quinizarin at 470 nm.

**Figure A2:**
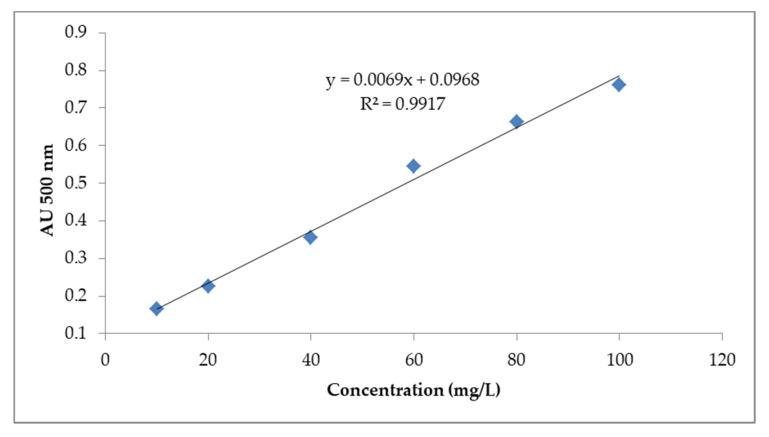
Standard curve for red yeast rice pigment (RYrp) at 500 nm.

Five milliliters of the fermented culture broth were sampled at predefined regular intervals and filtered through pre-weighed nylon mesh of 48 µm pore size (Nitex 07401, Sefar AG, Heiden, Switzerland) to separate the mycelia (Table 1). The concentration of extracellular pigments was determined using UV-visible spectrophotometer (UV-VIS spectrophotometer UV-1800, Shimadzu, Tokyo, Japan) at 470 (orange pigments) and 500 nm (red pigments), as the color in the extracellular culture filtrate of *T. albobiverticillius* 30548 was intense dark red [39]. Quinizarin (Sigma-Aldrich, St. Louis, MO, USA) and Red Yeast rice pigments (RYrp) (Wuhan Jiacheng Biotechnology Co., Ltd., Wuhan, China) were used as the reference standards, since the color and absorbance profile of the pigments produced by *T. albobiverticillius* 30548 were similar to those of these two compounds, at a wavelength of 470 and 500 nm. Thus, the OD values at 470 and 500 nm were interpolated using the standard curve equations of the two chosen standards, respectively (Figure A1 and Figure A2 in Appendix A), to obtain the pigment concentration in terms of gram per liter equivalents (*g/L* equivalent). The concentrations of red and orange pigments (*g/L* equivalent) were consequently calculated using Equations (1) and (2), as shown below:

(1) $$\mathrm{Orange}\;\mathrm{pigment}\;\mathrm{concentration},\;C\;\left(\frac{g}{L}equiv.\right)=\frac{Abs_{470\;}-0.1102}{0.0148}*0.001,$$

(2) $$\mathrm{Red}\;\mathrm{pigment}\;\mathrm{concentration},\;C\;\left(\frac{g}{L}equiv.\right)=\frac{Abs_{500\;}-0.0968}{0.0069}*0.001,$$

### 2.5. Dry Biomass Concentration

The amount of wet biomass was obtained through filtration using 48 m Nitex filter cloth (SEFAR AG, Switzerland) and was weighed precisely using an analytical balance. The filters with wet biomass was dried in a hot air oven (SNB 100, Memmert GMBH, Schwabach, Germany) at 105 °C for 17 h. The dried filters were weighed after letting them cool in a desiccator for 30 min to bring it down to room temperature. The amount of dry biomass in the culture broth was inferred from the following, Equation (3):

(3) $$\;[C_{DB}]\frac{g}{L}=\frac{\left(W2-W_{1}\right)}{V\;},$$

where W_2_ is the weight of the filter with biomass obtained after drying (*g*), W_1_ is the weight of the corresponding empty filter (*g*), and V is the volume of sample (*L*).

### 2.6. Experimental Design and Statistical Analysis (BBED and RSM) 

Box–Behnken experimental design (BBED) and response surface modeling (RSM) with four factors (pH, temperature, agitation speed, and fermentation time) at three levels were used in this study to investigate and optimize the effect of process parameters on the production of pigments (orange and red pigments) and fungal biomass. The process parameters and their ranges were chosen, based on preliminary “one-factor-at-a-time” studies (Table 1) (data not shown). Each independent variable was coded at three levels as −1 (low), 0 (central point), and +1 (high), which were—pH (4–9), temperature (21–27 °C), agitation speed (100–200 rpm), and fermentation time (24–336 h). The process variables were denoted as *X_1_*, *X_2_*, *X_3_*, and *X_4_* and the actual value of process parameters were converted into the uncoded form, using the following Equation (4) [40].

(4) $$x=\frac{X-((X_{\max}+X_{\min})/2)}{(X_{\max}-X_{\min})/2},$$

In total, twenty-nine experiments were carried out including five center points. Design-Expert^®^ Software (Version 9, Stat-Ease Inc., Minneapolis, MN, USA) was used in this study to construct the experimental design and statistically analyze the experimental data.

The obtained experimental results should fit well into the empirical second-order polynomial model (Equation (5)). The second-order polynomial equation correlated the relationship between independent variables and the responses. The mathematical form of the equation is given below:

(5) $$Y=\beta_{0}+\overset{k}{\underset{j=1}{\Sigma }}\beta_{j}x_{j}+\overset{k}{\underset{j=1}{\Sigma }}\beta_{jj}x_{j}^{2}+\underset{i}{\Sigma }\overset{k}{\underset{<j=2}{\Sigma }}\beta_{ij}x_{i}x_{j}+e_{i},$$

where Y is the response; *x_i_* and *x_j_* are variables (*i* and *j* range from 1 to *k*); *β_0_* is the model intercept coefficient; *β_j_*, *β_jj_* and *β_ij_* are interaction coefficients of the linear, quadratic, and second-order terms, respectively; *k* is the number of independent parameters (*k* = 4 in this study); and *e_i_* is the error [41,42,43].

The sequential model sum of squares and model summary statistics were carried out on the obtained experimental data to evaluate the adequacy of various models (linear, interactive, quadratic, and cubic) fitted to the experimental data. Pareto analysis of variance (ANOVA) was utilized in this present work in order to generate the ANOVA table. The experimental data were evaluated with various descriptive statistical analyses such as *p* value, F value, degrees of freedom (DF), coefficient of variation (CV), coefficient of determination (R^2^), adjusted coefficient of determination (Adj-R^2^), and predicted coefficient of determination (Pre-R^2^) to reveal the numerical consequence of the constructed quadratic mathematical model.

### 2.7. Optimization and Validation

The effect of fermentation process parameters on the responses, such as the pigment yields and the biomass were optimized by multi-response analysis, using Derringer’s desired function methodology [44]. In this method, the responses were transformed into a dimensionless individual desirability function (*d_i_*) that varied from 0 to 1 (lowest to highest desirability). From the geometric means of individual desires, the overall desirability function (*D*) was obtained using the following equation (Equation (6)).

(6) $$D={\left(d_{1}^{n_{1}}\times d_{2}^{n_{2}}\times d_{3}^{n_{3}}\times ...........\times d_{k}^{n_{k}}\right)}^{1/k}$$

where *d_i_* is the individual desirability ranged from 0 to 1, *k* is the number of considered responses, and *n_i_* is the weight of each response.

The dimensionless desirability (*d_i_*) value of the response using the maximized function was calculated from the equation below (Equation (7)).

(7) $$d_{i}=\frac{Y_{i}-Y_{min}}{Y_{max}-Y_{min}},$$

where, *Y_i_* is the obtained response value, *Y_min_* is the obtained response minimum value, and *Y_max_* is the obtained response maximum value.

To determine the validity of the developed mathematical model equation, triplicate experiments were performed under the optimal condition, as predicted by the model. The average values of the experimental data were compared with the predicted values of the developed model to find out the accuracy and suitability of this model.

## 3. Results

### 3.1. Box–Behnken Experimental Design Analysis

The selected statistical methods measured the effects of change in the operating variables and their mutual interactions on a system or a process through experimental design [45]. In this study, using the Design-Expert^®^ Software (v9), the Box–Behnken experimental design with four factors at three levels was employed to investigate and optimize the influence of the process variables on the yields of pigments (orange and red) and fungal biomass for *T. albobiverticillius* 30548. The results are listed in Table 2.

### 3.2. Development of Second-Order Polynomial Models 

Model adequacy checking was performed on the experimental data to determine whether the fitted model would give poor or misleading results. Four-degree polynomial models viz., linear, interactive (2 factors interaction, 2FI), quadratic and cubic models were fitted to the experimental data. Two different tests namely the sequential model sum of squares and model summary statistics were carried out in this study, to conclude the adequacy of models among various models to represent the responses. The adequacy of model summary output indicated that, the quadratic model was statistically highly significant due to its higher R^2^, compared to the other models (Table 3). Hence, the quadratic model was selected in this study to investigate the effect of the process variables on the responses, such as pigments and biomass production in the *T. albobiverticillius* 30548 strain.

### 3.3. Determination of Second-Order Polynomial Equations

An empirical relationship expressed by a second-order polynomial equation with interaction terms was fitted between the experimental results obtained on the basis of the Box–Behnken experimental design and the input variables. The observed experimental results in each run were subjected to multiple regression analysis to calculate the regression coefficients of the model. Calculated regression coefficients were substituted in (Equation (4)) to obtain a model for the orange pigment yield OPY (Equation (8)), red pigment yield RPY (Equation (9)), and dry biomass weight DBW (Equation (10)). The final equations obtained in terms of the coded factors are given below:

(8) $$OPY=+1.74+0.35X_{1}-0.11X_{2}+0.27X_{3}-0.029X_{4}-0.11X_{1}X_{2}-0.19X_{1}X_{3}+0.11X_{1}X_{4}+0.41\;X_{2}X_{3}+2.145^{-17}\;X_{2}X_{4}+0.10X_{3}X_{4}-0.42X_{1}^{2}-0.68X_{2}^{2}-0.35X_{3}^{2}-0.38X_{4}^{2},$$

(9) $$RPY=+1.92+0.28X_{1}-0.09X_{2}+0.20X_{3}-0.015X_{4}-0.087X_{1}X_{2}-0.17X_{1}X_{3}+0.075X_{1}X_{4}+0.31\;X_{2}X_{3}-0.019X_{2}X_{4}+0.071X_{3}X_{4}-0.33X_{1}^{2}-0.54X_{2}^{2}-0.31X_{3}^{2}-0.32X_{4}^{2},$$

(10) $$DBW=+6.80+0.77X_{1}-0.77X_{2}+1.24X_{3}-0.17X_{4}-0.24X_{1}X_{2}-0.021X_{1}X_{3}-0.61X_{1}X_{4}+1.18\;X_{2}X_{3}+055X_{2}X_{4}+0.17X_{3}X_{4}-1.02X_{1}^{2}-2.31X_{2}^{2}-1.35X_{3}^{2}-0.97X_{4}^{2},$$

where *X_1_*, *X_2_*, *X_3_*, and *X_4_* are the coded values of the independent process variables, respectively, pH, temperature, agitation speed, and fermentation time.

### 3.4. Statistical Analysis

The adequacy and fitness of the models were tested by multiple regression analysis using the least square method. Significance of the developed models could be determined through ANOVA and the results are shown in Table A1 (Appendix B). The results of ANOVA indicated that, the developed models adequately represented the actual relationship between the independent variables and responses, in the chosen range. Analysis of variance followed by Fisher’s statistical test (F-test) was applied to evaluate the significance of each variable. The high F- values, 67.05 for the orange pigment yield (OPY) measured at 470 nm, 36.61 for red pigment yield (RPY) measured at 500 nm, and 34.02 for the dry biomass weight (DBW) indicated that most of the variation in the response could be explained by the developed regression equations. The associated *p*-values were used to estimate whether F is large enough to indicate statistical significance, and *p*-values lower than 0.05 indicated that the developed model and the terms were statistically significant. In our study, the *p*-values were lower than 0.0001 for all responses (OPY, RPY, DBW). It exhibited the precision and the accuracy of the developed models.

Determination coefficient (R^2^), adjusted R^2^ (AdjR^2^), predicted R^2^ (PreR^2^), and coefficient of variation (CV%) were calculated to check the adequacy (Adeq. Pre.) and accuracy of the developed models. The R^2^ gave the proportion of total variation in the responses predicted by the models. The values of R^2^ (0.9853 for OPY, 0.9734 for RPY, and 0.9714 for DBW) ensured a satisfactory fit of the quadratic model to the experimental data. For example, R^2^ values indicated that the sample variation of 98.5% for OPY was attributed to the independent variables and only 1.5% of the total variations were not explained by the model. The adjusted determination coefficient (AdjR^2^) corrected the R^2^ value of the sample size and the number of terms in the model. In this study, the values of AdjR^2^ (0.9706 for OPY at 470 nm, 0.9468 for RPY at 500 nm, and 0.942 for DBW) were also high and very close to the R^2^ values, and indicated a better prediction of the model. However, when irrelevant variables are added to the model, the adjusted R^2^ decreases. In general, AdjR^2^ will always be less than or equal to R^2^. PreR^2^ is a measure of how good the model predicts a response value. In our case, the PreR^2^ (0.9172 for OPY at 470 nm, 0.8469 for RPY at 500 nm, and 0.8445 for DBW) are in reasonable agreement with the AdjR^2^.

The CV%, indicating the relative dispersion of the experimental points from the predictions of the second-order polynomial (SOP) models, were found to be 9.12 for OPY at 470 nm, 7.41 for RPY at 500 nm, and 9.41 for DBW, respectively. The low values of CV% clearly indicated a very high degree of precision and a good reliability of the experimental values. The high R^2^ value and a small CV% value indicated that the developed model would be able to give a good estimate of response of the system over the ranges studied.

### 3.5. Effect of Process Variables on Pigment Yield

In our study, the growth of the fungus in PDB leads to the production of secondary metabolites, which are visualized through the formation of a colored broth during the fermentation process. Production of pigments started at 48 h, attained a maximum at 140 h, and showed a slight increase, thereafter, until the end of fermentation [39,46]. In pigment production, temperature and pH acts as a whole active mechanism that is probably related to the genetic and metabolic control of defense mechanisms, thus influencing the level of pigment concentration and high productivity of pigments [47].

#### 3.5.1. Combined Effect of pH, Temperature, Agitation Speed, and Time on Orange Pigment Yield (OPY) 

The effect of independent variables on orange pigment yield was studied by changing the levels of any two independent variables, while keeping the other two at their constant middle level. The response surface plots (RSM) were used to locate the optimal values for this fermentation process. Therefore, three response surface plots were obtained by considering all possible combinations.

**Figure 1:**
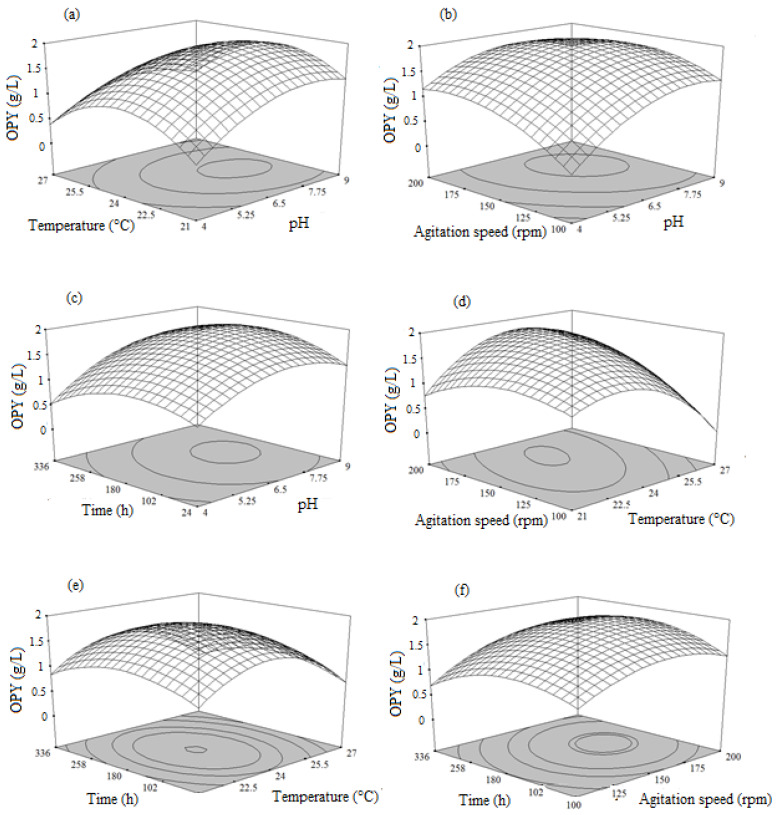
Response surface plots showing the influence of the process variables on the orange pigment yield (OPY): (**a**) Interaction between temperature and pH; (**b**) Interaction between agitation speed and pH; (**c**) Interaction between time and pH; (**d**) Interaction between agitation speed and temperature; (**e**) Interaction between time and temperature; (**f**) Interaction between time and agitation speed.

Figure 1a shows the effect of interaction between pH and temperature on the orange pigment production (maximum absorbance at 470 nm). It revealed that both factors at their lower levels had a negative impact on the orange pigment production. Increase in pH and temperature led to a gradual increase in pigment production, of up to pH 7.5 and temperature 24 °C. Increasing the value of both independent variables (pH above 7.8 and temperature above 27 °C) also showed a negative effect on orange pigment production. As shown in Figure 1b, increasing the agitation speed (up to 155 rpm) and pH (up to 7.5) led to high pigment production (1.76 g/L of orange pigments quinizarin equivalent), while agitation speed above 155 rpm and pH above 7.5 showed a negative effect on orange pigment production.

The interaction between fermentation time and pH also play an important role in pigment production (Figure 1c); pH in the range of up to 8.1 (alkaline pH) at higher levels of fermentation time, was found to be significant for orange pigment production. It was noticed that fermentation time (234 h) and pH (<8.1) up to a certain level supported orange pigment production but both factors negatively applied at higher levels and affected the pigment production. Temperature and agitation speed showed significant influence on each other and also on OPY. Both parameters showed a linear and quadratic response for pigment yield (Figure 1d). Maximum yield was observed in the above mid-values for both parameters (temperature of 25 °C and agitation speed of 165 rpm) and above that level, yields of the pigment decreased. The interactive effects of temperature and fermentation time against OPY are depicted in Figure 1e. When temperature increased from 21 to 24 °C and fermentation time was between 24 and 259 h, it resulted in a gradual increase in pigment production, up to a maximum. Further increase in temperature (above 24 °C) and fermentation time (above 259 h) led to a decrease in pigment yield. 

The three-dimensional (3D) response interactive plot of agitation speed and fermentation time illustrated that, OPY had increased with increase in agitation speed and fermentation time, up to 180 rpm and 224 h, respectively (Figure 1f). The yield of orange pigment was decreased with an increase in the agitation speed and fermentation time above 180 rpm and 247 h, respectively.

Combining the effects of all process variables investigated in this work, the initial pH and temperature of the medium played a key role in orange pigment synthesis. It was reported that a more acidic pH is favorable for most fungi for pigment synthesis in a submerged culture [4,48,49]. Similarly, acidic pH around 6.5 combined with temperature of up to 23.9 °C, agitation speed of 154.9 rpm, and fermentation time of 229 h was considered to be the optimal condition to produce maximal OPY of 1.76 g/L quinizarin equivalent.

#### 3.5.2. Combined Effects of pH, Temperature, Agitation Speed, and Time on Red Pigment Yield (RPY)

**Figure 2:**
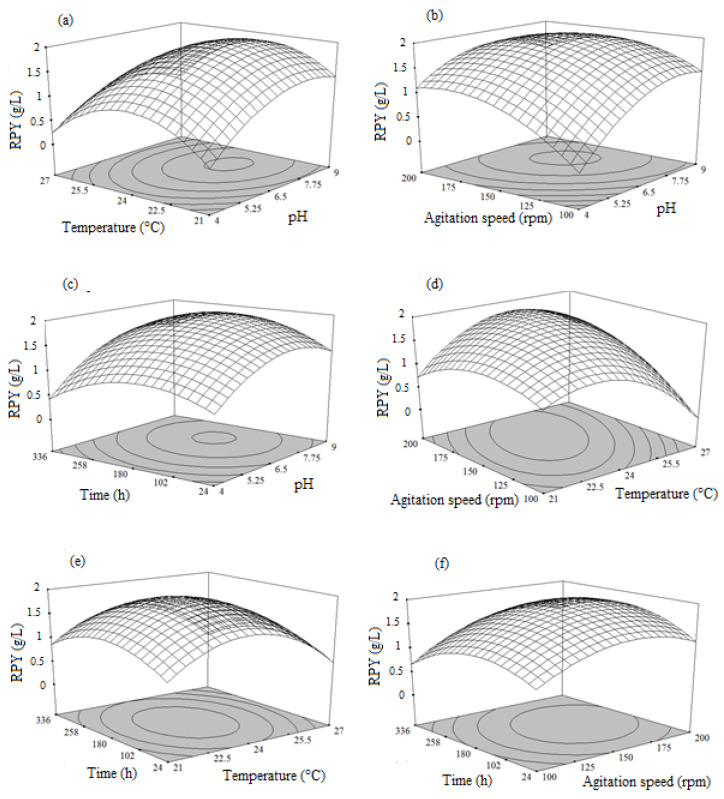
Response surface plots showing the influence of the process variables on the red pigment yield (RPY): (**a**) Interaction between temperature and pH; (**b**) Interaction between agitation speed and pH; (**c**) Interaction between time and pH; (**d**) Interaction between agitation speed and temperature; (**e**) Interaction between time and temperature; (**f**) Interaction between time and agitation speed.

Data obtained from the experiments were used to study the effect of process variables on red pigment production. From the results, it was confirmed that increasing pH and temperature from 4.0–6.5 and 22–25 °C enhanced the intensity of red pigment production in *T. albobiverticillius* 30548 and then decreased it (Figure 2a).

Additionally, increasing the agitation speed from 100–158 rpm and the pH value from 4.0–6.5 enhanced the red pigment production (Figure 2b). The maximum production of red pigments was attained at 120–168 h of fermentation and further increase in incubation time led to a decrease in the production (Figure 2c,e,f). The mycelium of the species grew rapidly at the start of fermentation (12–48 h), matured in the period of 48–96 h, and more extracellular red metabolites were released for up to 168 h, in the fermentation medium. Further increasing incubation time led to the bleaching of the extracellular red color and then a decrease in the pigment yield. To examine the influence of agitation speed on red pigment production, experiments were carried out in various agitation speeds (100–200 rpm). The results showed that, red pigment production increased from 100–158 rpm and above that, it decreased rapidly (Figure 2d,f). 

Maximum pigment production was exhibited in the middle level of the process variables and strong interaction was found between the variables, since the shape of the response surface and contour plot was elliptical in nature. By applying the numerical optimization method, the optimal condition was attained as follows—initial pH of 6.5, temperature of 24 °C, agitation speed of 158.4 rpm, and fermentation time of 198.6 h, with a maximal red pigment yield of 1.92 g/L, in terms of RYrp equivalent, respectively.

#### 3.5.3. Combined Effects of pH, Temperature, Agitation Speed, and Time on Dry Biomass Weight (DBW)

**Figure 3:**
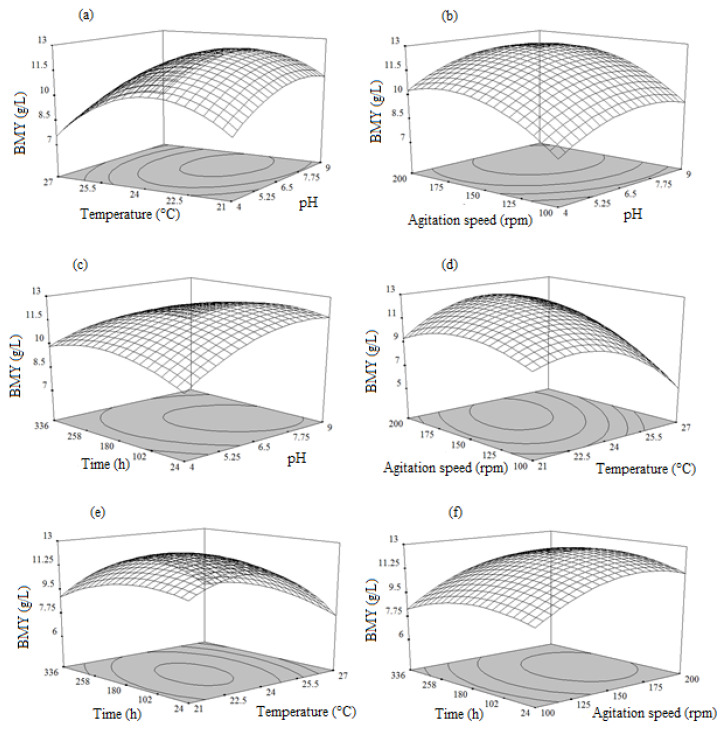
Response surface plots showing the influence of the process variables on dry biomass yield: (**a**) Interaction between temperature and pH; (**b**) Interaction between agitation speed and pH; (**c**) Interaction between time and pH; (**d**) Interaction between agitation speed and temperature; (**e**) Interaction between time and temperature; (**f**) Interaction between time and agitation speed.

The influence of the process variables over the dry biomass weight was similarly examined and the outcomes were depicted in Figure 3. The graph (Figure 3a) demonstrated the biomass yield was linearly increased with increasing levels of pH up to 7.8 and temperature up to 25 °C, and then the yields decreased gradually.

Figure 3b shows the effect of interaction between pH and agitation speed on biomass production, which revealed that both factors at their lower level had no significant effect on the biomass production, but increase in pH (up to pH 7.7) and agitation speed (up to 180 rpm) led to a gradual increase in the biomass production, while increase in the value of both independent variables beyond pH 7.7 and agitation speed of 180 rpm showed a negative effect on biomass production. From Figure 3c, it can be seen that the biomass yield increased with increasing pH and fermentation time, up to its middle level. However, further increasing pH and fermentation time would decrease the biomass yield. The 3D response surface plots based on independent variables such as temperature and agitation speed were developed, while other variables were kept at middle levels (Figure 3d) and exhibited that, the biomass yield was enhanced with increasing temperature (24.5 °C) and agitation speed (185 rpm), and the yield decreased beyond these levels. Biomass production was also increased with increase in both variables (temperature (up to 25 °C) and fermentation time (up to 245 h)) (Figure 3e). However, there was a sharp convergence of the curve near the boundary, explaining that in the presence of temperature and fermentation time above certain limit (above temperature of 25 °C and fermentation time of 245 h), it would not contribute to increasing biomass production. 

The interaction between agitation speed and fermentation time also played an important role in biomass production (Figure 3f). Agitation speed and fermentation time at above middle level was found to be significant for biomass production. It was noticed that agitation speed up to a certain level (190 rpm), as well as fermentation time (245 h) supported the biomass production but both affected the biomass production negatively, at higher levels. The analysis of response surface was performed in order to determine the optimal condition to produce maximal biomass yield. 

The optimal condition was an initial pH of 6.6, temperature of 23.9 °C, agitation speed of 168.9 rpm, and fermentation time of 145.1 h, respectively, and the maximum predicted yield of biomass (7.16 g/L) was attained in the optimal condition.

### 3.6. Multi Response Optimization

The second-order polynomial models developed in this study were utilized for each response, in order to obtain specified optimum conditions. An optimal condition for the maximum pigment production (orange and red) and biomass yield in *T. albobiverticillius* 30548 under submerged fermentation was derived by Derringer’s desired function methodology. This function searches for a combination of factor levels that simultaneously satisfies the requirements for each response in the design. Individual desirability (d_i_) evaluates how the process parameters optimize a single response. This numerical optimization evaluates a point that maximizes the desirability function. One main objective of optimization could be to maximize the final pigment yield and biomass production and recalculating all responsible independent factors by using desirability functions, therefore, the goal for pH, temperature, agitation speed, and fermentation time was assigned, as in range. 

The goal for the orange pigment yield (OPY), red pigment yield (RPY), and biomass production (DBW) was assigned to get the maximum. A weight factor of 1 was chosen for all individual desirability in this work. The “importance” of a goal could be changed in relation to the other goals. It could range from 1 (least importance) to 5 (most important). The default is for all goals to be equally important in a setting of 3. The optimization procedure was conducted under these settings and boundaries. Under the optimal conditions, the predicted orange pigment yield was 1.76 g/L quinizarin equivalent, red pigment yield was 1.92 g/L RYrp equivalent, and dry biomass yield was 7.16 g/L, with a desirability value of 0.983. The maximized overall desirability (D = 0.983) was calculated from the geometric means of the individual desirability functions (d_i_) of each response.

### 3.7. Validation of the Optimized Condition

**Figure 4:**
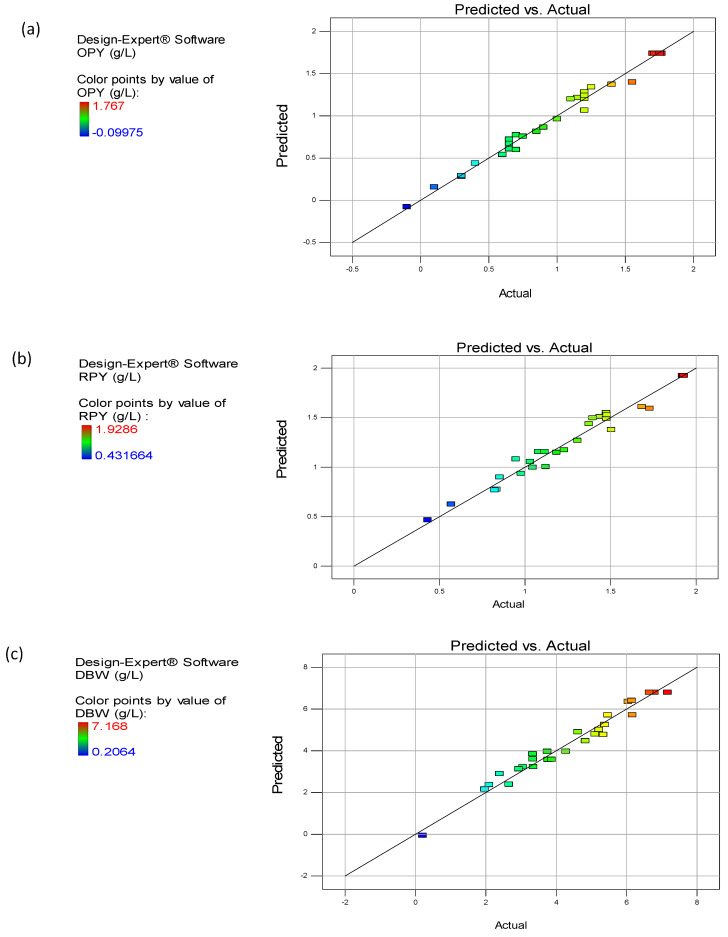
Validation of the polynomial model for (**a**) OPY, (**b**) red pigment yield (RPY), and (**c**) dry biomass weight (DBW) productions from *Talaromyces albobiverticillius* 30548.

The validation was carried out in shake flasks in triplicates under the optimized conditions of the media, predicted by the polynomial model. The mean of experimental values of OPY, RPY, and DBW were compared with the predicted values. The existence of a good correlation between the experimental and predicted values indicated the reliability and validity of the proposed model (Figure 4a–c). Due to the applicability of optimal extraction condition in a practical manner, the attained optimal condition was altered as follows—initial pH of 6.4, temperature of 24 °C, agitation speed of 164 rpm, and fermentation time of 149 h, respectively. The experimental efficiency of the production of pigments and dry biomass under the optimum condition was found to be 1.76 ± 0.58 g/L quinizarin equivalent for OPY, 1.92 ± 0.76 g/L RYrp equivalent for RPY, and 7.18 ± 0.41 g/L for DBW, respectively.

## 4. Discussion

The results obtained in this work for the cultivation of *T. albobiverticillius* 30548 were consistent with Babitha et al. (2007), who reported that maximum production of pigments was noticed in *M. purpureus* at pH 4.5 to 7.5 [50]. Studies by many researchers have revealed that among the numerous environmental factors, medium pH and the source of nitrogen determine pigment production in a submerged culture. The optimal pH for *T. albobiverticillius* 30548 was found to be 6.4 in this study, which is close to the optimum pH for pigment production in *Talaromyces verruculosus* mentioned by Chadni et al. (2017) [51]. Indeed, acidic pH might enhance the hydrolysis of the substrates and subsequently favors the generation of the metabolite production [52]. From Yongsmith et al. (1993) it was demonstrated that the compounds excreted in the culture broth could react with ammonium ion or free amino groups to transform to red amine derivatives towards neutral pH and beyond [53]. This might happen in *T. albobiverticillius* 30548 as there is an evolution of the color hue in the culture broth (from yellow to red), along the entire fermentation period [39].

Temperature, another key parameter, controls the growth and metabolic activity of fungi. To find the optimal temperature for mycelial growth and pigment production, *T. albobiverticillius* 30548 was cultivated under various temperatures (21–27 °C). Fermentation at 24 °C is regarded as favorable for fungal growth in combination with an acidic pH and a medium agitation speed, which aid good production of pigments. Mendez et al. (2011) found a similar result in obtaining the highest level of pigment production at 24 °C in *P. purpurogenum GH2*, combined with an acidic pH [28]. However, in other studies with *Monascus spp., Penicillium spp.,* temperature at 30 °C is considered to be the best temperature for red pigment production. However, the microbial growth was hindered at 30 °C but a maximum growth was attained by increasing the temperature to 34 °C [26,54,55]. For the marine-derived *T. albobiverticillius* 30548, it is interesting to note that the optimal temperature for both mycelial growth and pigment production was found to be 24 °C.

Fermentation of any substrate is influenced by the time of incubation, so the pigment synthesis was monitored at predefined intervals. Pigment production, as measured by absorbance, was slightly delayed when compared to biomass growth, and was higher at 149 h (day 6) of fermentation time, until the stationary phase [39,46]. After 149 h, a decrease in pigment production was observed, which might be due to the decomposition of pigments (degradation of the chromophore pigment group) or changes in the pigment structure. A similar outcome was observed in the pigment production by *Monascus purpureus* [56].

Basically, in liquid fermentation, agitation speed increases the amount of dissolved oxygen and makes the oxygen more accessible to cells, leading to greater growth [57]. Similarly, in this present work, increasing agitation speed from 100–158 rpm enhanced the supply of oxygen in the growth phase and thus enhanced the biomass and pigment yield. However, above 158 rpm, agitation speed might damage the mycelial growth by high shear force and lead to a decrease in the pigment yields. Mohamed et al. (2012) reported similar results, as the highest pigment production was obtained at agitation speed of 150 rpm using *Monascus purpureus* [58]. Additionally, the highest level of yellow pigments (1.38 g/L) was produced by *Penicillium aculeatum* ATCC 10409 at an agitation speed of 100 and 150 rpm [47].

## 5. Conclusions

Box–Behnken experimental design (BBED) coupled with response surface modeling (RSM) was successfully applied in the present work. The methodology allowed to locate the optimal process parameters in the chosen range (initial pH (4–9), temperature (21–27 °C), agitation speed (100–200 rpm), and fermentation time (0–336 h)), to maximize pigment and biomass production in submerged fermentation of *T. albobiverticillius* 30548. The outcomes of this study indicated that, all process variables had a significant influence on the responses. The actual values obtained through the experimental studies were used to construct second-order quadratic model for the responses to predict the observed data. The optimal condition was attained through Derringer’s desired function methodology (initial pH of 6.4, temperature of 24 °C, agitation speed of 164 rpm, and fermentation time of 149 h). It was materially validated by the experiment (1.76 ± 0.58 g/L quinizarin equivalent for OPY, 1.92 ± 0.76 g/L RYrp equivalent for RPY, and 7.18 ± 0.41 g/L for DBW).

The results of this study revealed that *T. albobiverticillius* 30548 could be an avenue for industrial application of pigment production. Considering the potential toxicity of compounds produced by *T. albobiverticillius* 30548 in SmF, complementary studies need to be conducted, based on the characterization of the 12 major compounds synthetized [59], and on the elucidation of the biosynthetic pathway, before using them for applications. In addition, the mathematical models constructed in this study and the methodology applied could be useful for the selection of appropriate parameters for the production of pigments at a large scale.

This study allowed for the consideration of other factors of pigment production, besides the physical process factors. Generally, carbon and nitrogen sources in the culture medium play a major role in the growth and production of metabolites. Further work on optimizing various nutrient types and their concentration in the culture media is in progress and will be developed in a further study.

## Figures and Tables

**Table 1:** Coded and actual values of the variables for the four factor Box–Behnken experimental design.

Variables	Symbol	Coded and Actual Values		
		−1	0	+1
pH	X_1_	4	6.5	9
Temperature (°C)	X_2_	21	24	27
Agitation speed (rpm)	X_3_	100	150	200
Fermentation time (h)	X_4_	24	168	336

**Table 2:** Box–Behnken design matrix with the experimental responses over process parameters in *Talaromyces albobiverticillius* 30548.

Exp. Run	pH	Temperature (°C)	Agitation Speed (rpm)	Fermentation Time (h)	Orange Pigment Yield (g/L^1^)	Red Pigment Yield (g/L^2^)	Dry Biomass Weight (g/L)
1	0	0	0	0	0.30	0.83	3.35
2	0	0	1	−1	1.15	1.44	5.38
3	0	1	0	1	0.30	0.82	1.97
4	−1	0	0	1	0.70	1.08	3.05
5	0	−1	0	−1	0.90	1.23	3.76
6	0	1	1	0	1.10	1.37	6.17
7	−1	0	0	−1	0.65	1.04	2.39
8	0	0	0	0	1.25	1.47	5.47
9	0	0	0	0	0.65	0.95	3.34
10	0	1	−1	0	1.20	1.48	6.05
11	1	1	0	0	0.40	0.85	4.83
12	0	0	0	0	1.40	1.68	5.10
13	0	0	0	0	1.00	1.31	3.33
14	0	−1	−1	0	−0.10	0.43	0.21
15	−1	0	1	0	0.65	1.03	3.75
16	−1	−1	0	0	1.20	1.39	5.34
17	0	1	0	−1	0.10	0.57	2.66
18	1	0	−1	0	1.20	1.48	4.28
19	0	−1	1	0	1.20	1.50	4.61
20	1	0	1	0	1.55	1.73	6.15
21	1	0	0	−1	0.85	1.18	5.21
22	0	−1	0	1	0.70	1.12	2.10
23	0	0	−1	−1	0.75	1.12	3.87
24	1	−1	0	0	0.60	0.98	2.93
25	−1	1	0	0	1.70	1.92	6.67
26	1	0	0	1	1.71	1.92	6.7
27	−1	0	−1	0	1.76	1.93	6.79
28	0	0	1	1	1.77	1.93	7.17
29	0	0	−1	1	1.75	1.93	6.65

1: g/L equiv. quinizarin 2: g/L equiv. RYrp.

**Table 3:** Model summary statistics for responses.

Source	Std. Dev.	R^2^	Adjusted R^2^	Predicted R^2^	Press	Remarks
Model summary statistics for Orange Pigment Yield						
Linear	0.46	0.3306	0.2191	0.1387	6.54	
2FI	0.48	0.4574	0.1560	0.0518	7.20	
Quadratic	0.089	0.9853	0.9706	0.9172	0.63	Suggested
Cubic	0.072	0.9959	0.9809	0.4736	4.00	Aliased
Model summary statistics for Red Pigment Yield						
Linear	0.37	0.3146	0.2004	0.1230	4.29	
2FI	0.39	0.4328	0.1178	0.0127	4.82	
Quadratic	0.096	0.9734	0.9468	0.8469	0.75	Suggested
Cubic	0.058	0.9958	0.9806	0.402	2.92	Aliased
Model summary statistics for Dry Biomass Weight						
Linear	1.49	0.3828	0.2799	0.1902	69.91	
2FI	1.58	0.4820	0.1943	−0.0036	86.64	
Quadratic	0.42	0.9714	0.9429	0.8445	13.42	Suggested
Cubic	0.18	0.9977	0.9894	0.9790	1.81	Aliased

## Appendix B

**Table A1:** Regression coefficient (RC) of the models and their statistical parameters on the responses (Xi: coded values of independent variables (i = 1: pH; i = 2: temperature, i = 3: agitation speed; i = 4: fermentation time) and their interactions.

Source	DF	Orange Pigment Yield		Red Pigment Yield		Dry Biomass Weight	
		RC	*p* Value *	RC	*p* Value *	RC	*p* Value *
Model	14	1.74	<0.0001	1.92	<0.0001	6.80	<0.0001
X_1_	1	0.35	<0.0001	0.28	<0.0001	0.77	<0.0001
X_2_	1	−0.11	0.0009	−0.090	0.0058	−0.77	<0.0001
X_3_	1	0.27	<0.0001	0.20	<0.0001	1.24	<0.0001
X_4_	1	−0.029	0.2767	−0.015	0.5901	−0.17	0.1837
X_12_	1	−0.11	0.0245	−0.087	0.0927	−0.24	0.2788
X_13_	1	−0.19	0.0009	−0.17	0.0031	−0.02	0.9224
X_14_	1	0.11	0.0245	0.075	0.1394	−0.61	0.0117
X_23_	1	0.41	<0.0001	0.31	<0.0001	1.18	<0.0001
X_24_	1	0.000	1.000	−0.019	0.7011	0.55	0.0210
X_34_	1	0.10	0.0418	0.071	0.1615	0.17	0.4359
X_1_^2^	1	−0.42	<0.0001	−0.33	<0.0001	−1.02	<0.0001
X_2_^2^	1	−0.68	<0.0001	−0.54	<0.0001	−2.31	<0.0001
X_3_^2^	1	−0.35	<0.0001	−0.31	<0.0001	−1.35	<0.0001
X_4_^2^	1	−0.38	<0.0001	−0.32	<0.0001	−0.97	<0.0001
R^2^		0.9853		0.9734		0.9714	
AdjR^2^		0.9706		0.9468		0.9429	
PreR^2^		0.9172		0.8469		0.8445	
CV%		9.12		7.41		9.41	
Adeq. Pre.		28.30		21.04		22.71	

R^2^: Determination coefficient; AdjR^2^: adjusted R^2;^ PreR^2^: predicted R^2^; CV%: coefficient of variation; and Adeq. Pre.: predicted adequacy. *p* < 0.01 highly significant; 0.01 < *p* < 0.05 significant; and *p* > 0.05 not significant.
